# Challenging the roles of CD44 and lipolysis stimulated lipoprotein receptor in conveying *Clostridium perfringens* iota toxin cytotoxicity in breast cancer

**DOI:** 10.1186/1476-4598-13-163

**Published:** 2014-07-02

**Authors:** Katerina D Fagan-Solis, Denise K Reaves, M Cristina Rangel, Michel R Popoff, Bradley G Stiles, Jodie M Fleming

**Affiliations:** 1 Department of Biology, North Carolina Central University, Durham, NC, USA; 2 Tumor Growth Factor Section, Laboratory of Cancer Prevention, Frederick National Laboratory for Cancer Research, Frederick, MD, USA; 3 Institut Pasteur, Anaerobic Bacteria and Toxins Unit, Paris, France; 4 Department of Biology, Wilson College, Chambersburg, PA, USA

**Keywords:** *Clostridium perfringens* iota toxin, Lipolysis stimulated lipoprotein receptor, CD44, Breast cancer, Endocytosis, Cytotoxicity, Tamoxifen resistance

## Abstract

**Background:**

Translational exploration of bacterial toxins has come to the forefront of research given their potential as a chemotherapeutic tool. Studies in select tissues have demonstrated that *Clostridium perfringens* iota toxin binds to CD44 and lipolysis stimulated lipoprotein receptor (LSR) cell-surface proteins. We recently demonstrated that LSR expression correlates with estrogen receptor positive breast cancers and that LSR signaling directs aggressive, tumor-initiating cell behaviors. Herein, we identify the mechanisms of iota toxin cytotoxicity in a tissue-specific, breast cancer model with the ultimate goal of laying the foundation for using iota toxin as a targeted breast cancer therapy.

**Methods:**

*In vitro* model systems were used to determine the cytotoxic effect of iota toxin on breast cancer intrinsic subtypes. The use of overexpression and knockdown technologies confirmed the roles of LSR and CD44 in regulating iota toxin endocytosis and induction of cell death. Lastly, cytotoxicity assays were used to demonstrate the effect of iota toxin on a validated set of tamoxifen resistant breast cancer cell lines.

**Results:**

Treatment of 14 breast cancer cell lines revealed that LSR+/CD44- lines were highly sensitive, LSR+/CD44+ lines were slightly sensitive, and LSR-/CD44+ lines were resistant to iota cytotoxicity. Reduction in LSR expression resulted in a significant decrease in toxin sensitivity; however, overexpression of CD44 conveyed toxin resistance. CD44 overexpression was correlated with decreased toxin-stimulated lysosome formation and decreased cytosolic levels of iota toxin. These findings indicated that expression of CD44 drives iota toxin resistance through inhibition of endocytosis in breast cancer cells, a role not previously defined for CD44. Moreover, tamoxifen-resistant breast cancer cells exhibited robust expression of LSR and were highly sensitive to iota-induced cytotoxicity.

**Conclusions:**

Collectively, these data are the first to show that iota toxin has the potential to be an effective, targeted therapy for breast cancer.

## Background

Breast cancer is a heterogeneous disease that varies in etiology, pathophysiology and response to therapy. As a result, patients with disease of similar stage and grade often respond differently to therapy leading to disparate clinical outcomes. Molecular profiles characterizing the various intrinsic breast cancer subtypes, as per gene expression signatures, have been successful for predicting overall survival, relapse, and response to chemotherapy [1-4]. Luminal subtypes are defined by expression of estrogen receptor α (ERα) and cell cytokeratins (CKs) 8 and 18 [5,6]. Basal-like tumors are typically triple-negative (i.e. lacking expression of ERα, progesterone receptor, and human epidermal growth factor receptor 2 (HER2)), yet express basal CKs 5, 14, and/or 17 [5,7,8]. The claudin-low subtype is characterized by low gene expression of junction and adhesion proteins that include claudins 3, 4 and 7, as well as E-cadherin [3]. While these tumors initially respond to chemotherapy, there is a high risk of recurrence and disease progression, consequently leading to poor patient survival [9-11].

Abnormal protein regulation of cell-surface receptors promotes cancer development/progression, and is widely used to determine patient prognosis and dictate therapeutic regime. CD44 and lipolysis stimulated lipoprotein receptor (LSR) are both cell-surface, transmembrane proteins that mediate cellular responses towards their microenvironment. These molecules participate in cell-cell and cell-matrix interactions, as well as regulate cell growth, survival, differentiation, and motility [12-14]. High CD44 levels are a marker for tumor initiating and chemotherapeutic-resistant cells in many cancers, including breast [15,16]. High CD44-expressing cells have heightened tumorigenicity, self-renewal *in vivo,* and give rise to functional as well as molecular heterogeneity: properties directly linked to chemotherapeutic-resistant, aggressive cancers [15]. It has also been reported that basal-like tumors contain the highest percentage of CD44-positive cells [17], while high CD44 expression correlates to a basal-like phenotype, increased metastases, and unfavorable prognosis in breast cancer patients [18-20]. Similar to high CD44 levels, increased expression of LSR has been associated with altered gene expression of pathways involved in transformation and tumorigenesis, enhanced proliferation, survival in anchorage independent conditions and promotion of collective cell migration in breast cancer cells [21]. High LSR levels have also been identified as a marker for tumor-initiating and chemotherapeutic-resistant cells [14]. Collectively, these studies highlight a direct role for LSR in driving aggressive breast cancer behavior.

The use of bacterial toxins for selective and efficient cancer therapeutics has been gaining attention due to recent successes *in vitro* and *in vivo*[22,23]. Bacterial toxins possess efficient cytotoxic capabilities, making them suitable candidates for gene therapeutic applications towards various cancers [24-31]. *Clostridium perfringens* iota toxin has various properties that make it a potential candidate for targeted cancer therapy. For instance, like many of the “classic” AB exotoxins, iota toxin is secreted by the bacterium and contains two functionally distinct, subunits not linked in solution [32]. The B subunit (Ib) binds to a cell-surface receptor, facilitating docking and uptake of the enzymatic A subunit (Ia) through receptor-mediated endocytosis. Ib forms heptamers on the cell surface and creates pores within an acidified endosome membrane enabling release of Ia into the cytosol. The Ia molecule mono-ADP-ribosylates G actin, subsequently preventing F actin assembly that leads to overt rounding of cells and death [32-34].

Recent studies have implicated LSR and CD44 as functional receptors, or co-receptors, mediating iota toxin binding to host cells [35,36]. However, the relationship between these cell-surface proteins with respect to promotion of iota cytotoxicity is still unclear, given the various model systems used during these investigations. In the present study, we investigated the role of LSR and CD44 in a tissue-specific manner by identifying which protein mediates the cytotoxic processes specific to breast cancer. Ultimately, our future goal is to evaluate the potential of using iota toxin as an adjuvant, targeted therapy for breast cancers that may be less toxic than current available treatments.

## Results

### Relationship of LSR and CD44 expression levels in breast cancer subtypes to iota toxin sensitivity

**Figure 1:**
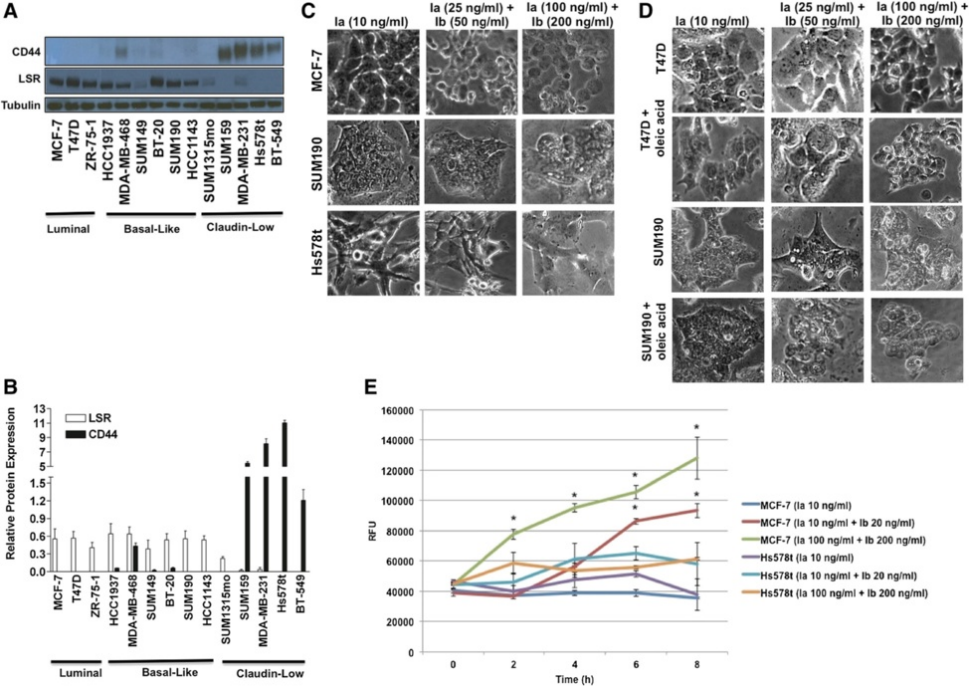
**Breast cancer cells expressing LSR are sensitive to iota toxin. (A)** Representative breast cancer cell lines were grown to 80% confluence, lysates isolated, and then analyzed via western blot using LSR and CD44 specific antibodies; α-tubulin was used as a loading control. Representative western blot and **(B)** corresponding intensity measured via ImageJ. Data represent mean relative intensity +/− SE. Cells were treated with Ia or Ia + Ib for 8 h in the absence **(C)** and presence **(D)** of 0.8 mM oleic acid in normal growth media for 20 min at 37°C prior to treatment. Representative images demonstrate cell rounding and detachment from tissue culture dish, indicative of cell death. **(E)** Cells were treated with Ia or Ia + Ib in complete growth medium at 37°C and cell death was quantified at the indicated times via fluorescent cytotoxicity assay. *P < 0.01. A minimum of three independent experiments was performed for each analysis. RFU; relative fluorescent units.

From the perspective of a protein-based treatment tool, *C. perfringens* iota toxin is relatively unexplored yet possesses promising potential for targeting breast cancer, as recent evidence now implicates LSR and CD44 as functional facilitators of iota cytotoxicity [35,36]. Thus, identifying the precise mechanisms of interaction between iota toxin with LSR and CD44 is a necessary step towards developing targeted therapies for breast cancer. Previous cancer studies have shown that LSR is positively correlated with ERα expression, tumor initiating cells, and chemoresistance [14,21]. Others have shown that high CD44 expression correlates to a basal-like phenotype and unfavorable prognosis in breast cancer patients [17-19]. Given this, we first assessed LSR and CD44 expression in a panel of well-characterized breast cancer cell lines. LSR was detectable in the majority of luminal and basal-like subtypes, while the claudin-low lines had little to no expression of LSR (Figure 1A,B). Conversely, CD44 expression was readily detectable in claudin-low lines while basal-like and luminal lines had little to below detectable expression levels via western blot analysis.

To further understand the role LSR and CD44 play in breast cancer susceptibility to iota toxin, we evaluated the effects of toxin exposure over time. Cells were treated with control (Ia only) or with varying concentrations of Ia and Ib as indicated (Figure 1C), under normal growth conditions. Toxin sensitivity, easily observed by robust cell rounding and altered morphology, was documented at 0, 1, 2, 4, 6, and 8 h post treatment. Results show that LSR+/CD44- lines were highly sensitive, LSR+/CD44+ lines were moderately sensitive, and LSR-/CD44+ lines were interestingly resistant to the cytotoxic effects of iota toxin in both a time and dose-dependent manner (Figure 1C; Table 1; cell rounding quantified in Additional file 1: Figure S1A). Furthermore, in fibroblasts and hepatocytes, binding of LSR to free fatty acids induces a conformational change that mediates binding of apoproteins B- and E-containing lipoproteins, leading to their subsequent internalization and degradation [37-39]. Thus, to further verify that iota toxin sensitivity is conveyed through LSR, we treated two LSR + cell lines moderately sensitive to iota toxin with oleic acid. Iota cytotoxicity was enhanced by oleic acid (Figure 1D; Table 2; cell rounding quantified in Additional file 1: Figure S1A).

**Table 1:** Iota toxin sensitivity in a breast cancer cell line panel

	**Cell line**	**Ia (10 ng/ml)**	**Ia (10 ng/ml) + Ib (20 ng/ml)**	**Ia (25 ng/ml) + Ib (50 ng/ml)**	**Ia (50 ng/ml) + Ib (100 ng/ml)**	**Ia (100 ng/ml) + Ib (200 ng/ml)**
**Luminal**	MCF-7	-	+	+	++	++
	T47D	-	+	+	+	++
	ZR-75-1	-	+	+	++	++
**Basal-like**	HCC1937	-	++	++	++	++
	MDA-MB-468	-	+	+	++	++
	SUM149	-	-	-	+	++
	BT-20	-	-	+	+	+
	SUM190	-	-	+	+	+
	HCC1143	-	++	++	++	++
**Claudin-low**	SUM1315mo	-	+	+	+	+
	SUM159	-	-	-	-	-
	MDA-MB-231	-	-	-	+	+
	Hs578T	-	-	-	-	-
	BT-549	-	-	-	-	-

(−) Resistant, 0 to 10% cell rounding; (+) Sensitive, 11 to 50% cell rounding; (++) Highly Sensitive, >50% cell rounding. Toxin Sensitivity recorded at 8 h post treatment.

**Table 2:** Iota toxin sensitivity of breast cancer cells following oleic acid treatment

**Cell line**	**Ia (10 ng/ml)**	**Ia (10 ng/ml) + Ib (20 ng/ml)**	**Ia (25 ng/ml) + Ib (50 ng/ml)**	**Ia (50 ng/ml) + Ib (100 ng/ml)**	**Ia (100 ng/ml) + Ib (200 ng/ml)**
T47D	-	+	+	+	++
T47D + Oleic acid	-	++	++	++	++
SUM190	-	-	+	+	+
SUM190 + Oleic acid	-	-	+	++	++

(−) Resistant, 0 to 10% cell rounding; (+) Sensitive, 11 to 50% cell rounding; (++) Highly Sensitive, >50% cell rounding. Toxin Sensitivity at 8 h post treatment.

To confirm the observed cell death, cytotoxicity assays were performed on highly sensitive, LSR+/CD44- MCF-7 and resistant LSR-/CD44+ Hs578t cells when treated with low and high concentrations of iota toxin (Figure 1E). Results indicated that highly sensitive MCF-7 cells had a significant increase in cytotoxicity in a time- and dose- dependent manner when challenged with iota toxin (*P* < 0.01). However, resistant Hs578t cells did not have significant changes in cytotoxicity compared to controls treated with Ia only (Figure 1E). No significant alteration in expression or activation (via cleavage) of cytochrome c or caspase-3 was detected via western blot analysis, suggesting that cell death was not mediated through these apoptotic pathways (*data not shown*). Collectively, these data suggest that expression of LSR and not CD44 is required for sensitivity of breast cancer cells to iota toxin.

### Glycosylation status of LSR and CD44 relative to iota toxin sensitivity

It is well established that glycosylation is necessary for proper functioning of CD44 in certain pathways [12,40-42]. While the glycosylation status of LSR is not characterized, sequence analysis indicates LSR contains residues as potential N-glycosylation sites. Thus, to determine whether glycosylation of LSR and/or CD44 plays a role in iota toxin sensitivity, we first assessed the glycosylation status of LSR. MCF-7 cells were treated with vehicle control or de-glycosylation agents Tunicamycin or Swainsonine (25 μg/ml, each). Western blot analysis indicated no shift in electrophoretic migration by SDS PAGE, suggesting that LSR was not heavily glycosylated (Additional file 2: Figure S2A). To verify that the de-glycosylating agents were functioning properly in the same cell line, expression of a known glycoprotein (E-cadherin) was assessed [43,44]. Treatment of MCF-7 cells with tunicamycin or swainsonine decreased the levels of glycosylated E-cadherin (top band) and increased the non-glycosylated (bottom) bands, thus confirming activity of the de-glycosylation agents (Additional file 2: Figure S2A). To confirm that the de-glycosylation agents did not affect the role of LSR cytotoxicity, LSR+/CD44- MCF-7 cells were cultured with or without Tunicamycin, followed by iota toxin. As shown in Additional file 2: Figure S2B and Additional file 3: Table S1, sensitivity of breast cancer cells to iota toxin was not altered by tunicamycin, suggesting that glycosylation does not play a role in toxin susceptibility.

Knowing that glycosylation is necessary for CD44 function in certain pathways [12,40-42], we sought to determine whether glycosylation was required to convey toxin resistance. When LSR-/CD44+ Hs578t cells were treated as described above, sensitivity to iota toxin was not altered, suggesting that deglycosylation of CD44 does not affect sensitivity to iota cytotoxicity (Additional file 2: Figure S2B and Additional file 3: Table S1).

Previous studies have determined that LSR and CD44 have multiple variants/isoforms that occur in cancer [45-47]. We performed variant specific qRT-PCR analysis in order to determine whether there was a correlation between iota toxin sensitivity and expression of LSR and/or CD44 variants. Although expression of LSR and CD44 variants varied among cell lines, there was no correlation between expression of LSR and/or CD44 variants with sensitivity to iota toxin (Additional file 4: Figure S3 and Additional file 5: Figure S4).

### LSR expression directly mediates iota toxin sensitivity in breast cancer cells

**Figure 2:**
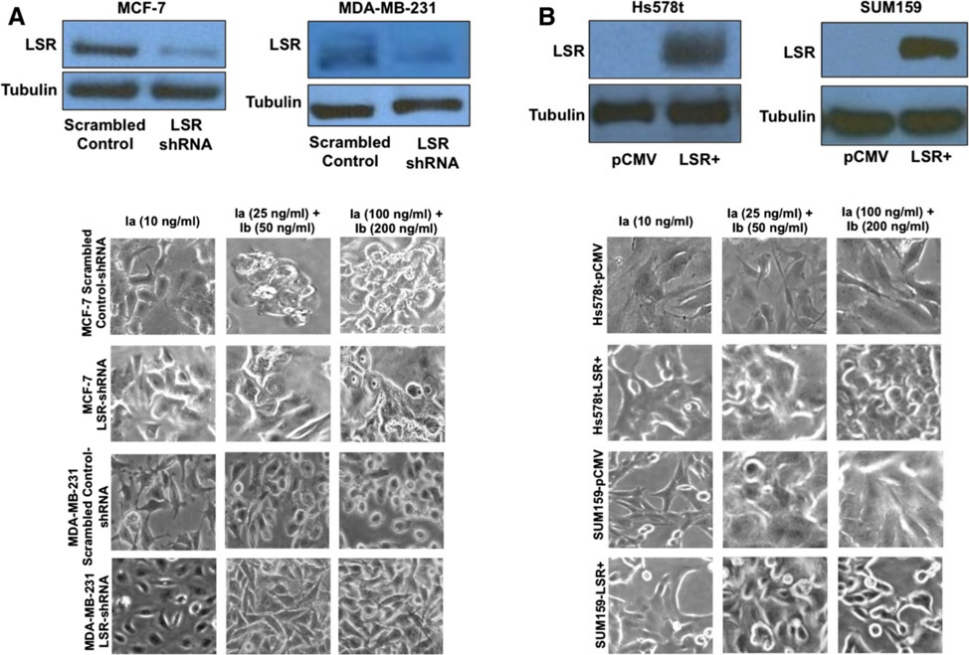
**Changes of LSR expression alter iota toxin sensitivity in breast cancer. (A)** MCF-7 and MDA-MB-231 cells were stably transfected with either a scrambled control shRNA plasmid (scrambled control-shRNA), or a plasmid containing shRNA specifically targeting LSR variant 1 (LSR-shRNA). Cells were grown to 80% confluence, lysates isolated, and then analyzed via western blot using an LSR-specific antibody and α-tubulin for loading control. Cells were treated with Ia or Ia + Ib for 8 h. Representative images demonstrate cell rounding and detachment from tissue culture dish, indicative of cell death. **(B)** Hs578t and SUM159 cells were stably transfected with either a control plasmid (pCMV), or a plasmid containing the full-length gene for LSR variant 1 (LSR+). Cells were grown and treated as stated in **(A)**. A minimum of three independent experiments was performed for each analysis.

To directly test the ability of LSR to convey iota toxin cytotoxicity, we stably knocked down LSR in MCF-7 and MDA-MB-231 cells resulting in decreased expression (64% and 46%, respectively) compared to control (Figure 2A). LSR knockdown lines were subjected to treatment with iota toxin, and as shown in Figure 2A and Table 3, reduced LSR expression in MCF-7 cells resulted in decreased sensitivity compared to scrambled control. This result could be due to the high expression of LSR in MCF-7 cells, suggesting there were still sufficient receptor numbers on the surface to facilitate intoxication. Conversely, knockdown of LSR in MDA-MB-231 cells, which have significantly lower basal levels of LSR expression (Figure 1A) resulted in a significant decrease in toxin sensitivity compared to scrambled, suggesting that reduction of LSR diminishes iota toxin sensitivity (Figure 2A; cell rounding quantified in Additional file 1: Figure S1B). To confirm our findings, LSR was overexpressed in two iota toxin resistant (LSR-/CD44+) cell lines, Hs578t and SUM159 (Figure 2B), and then treated with iota toxin. It is of note that overexpression of LSR in Hs578t and SUM159 cells did not increase sensitivity to iota toxin. While there were a few cells that succumbed to the toxin effects, the vast majority of the cell population remained resistant to the toxin (Figure 2B; Table 3; cell rounding quantified in Additional file 2: Figure S2B). These data suggested that introduction of LSR into LSR negative breast cancer cells does not increase sensitivity to iota toxin.

**Table 3:** Iota toxin sensitivity in LSR overexpressing and knockdown cells

	**Cell line**	**Ia (10 ng/ml)**	**Ia (10 ng/ml) + Ib (20 ng/ml)**	**Ia (25 ng/ml) + Ib (50 ng/ml)**	**Ia (50 ng/ml) + Ib (100 ng/ml)**	**Ia (100 ng/ml) + Ib (200 ng/ml)**
**Overexpressing**	SUM1315mo-CMV	-	-	-	-	+
	SUM1315mo-CD44+	-	-	-	-	-
	SUM1315mo-LSR+	-	-	+	+	++
	SUM159-CMV	-	-	-	-	-
	SUM159-LSR+	-	-	-	-	-
	Hs578t-CMV	-	-	-	-	-
	Hs578t-LSR+	-	-	-	+	+
**Knockdown**	MCF-7 Scrambled Control	-	+	+	++	++
	MCF-7 LSR shRNA	-	+	+	+	+
	SUM1315mo Control	-	+	+	+	+
	SUM1315mo-LSR shRNA	-	-	-	-	-
	MDA-MB-231 Scrambled Control	-	-	-	+	+
	MDA-MB-231 LSR shRNA	-	-	-	-	-

(−) Resistant, 0 to 10% cell rounding; (+) Sensitive, 11 to 50% cell rounding; (++) Highly Sensitive, >50% cell rounding. Toxin Sensitivity at 8 h post treatment.

### CD44 expression conveys resistance to iota toxin in breast cancer cells

**Figure 3:**
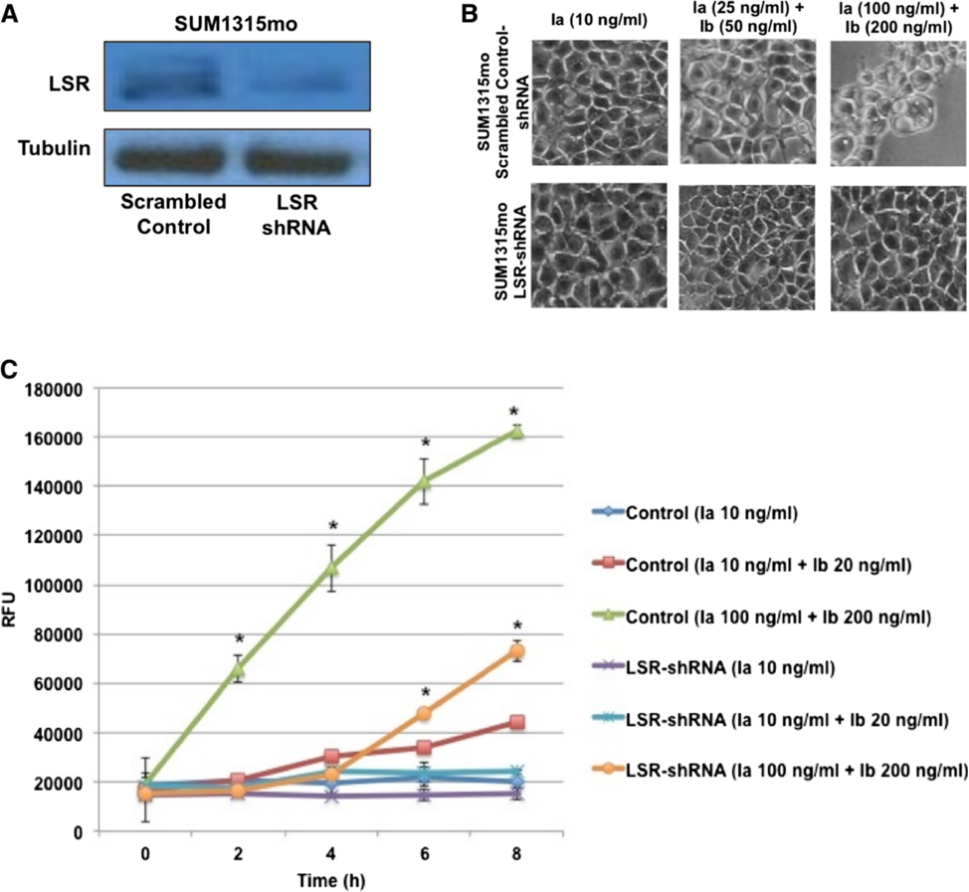
**Reduction of LSR expression increases resistance to cytotoxicity in LSR**^**low**^**/CD44**^**low **^**cells. (A)** SUM1315mo cells were stably transfected with either a scrambled control shRNA plasmid (scrambled control-shRNA), or a plasmid containing shRNA specifically targeting LSR variant 1 (LSR-shRNA). Lysates were isolated and analyzed via western blot using a LSR specific antibody and α-tubulin for loading control. **(B)** Cells were treated with Ia or Ia + Ib for 8 h in complete growth medium at 37°C. Representative images demonstrate cell rounding and detachment from tissue culture dish, indicative of cell death. **(C)** Cells death visualized in **(B)** was quantified at the indicated times via fluorescent cytotoxicity assay. *P < 0.05. A minimum of three independent experiments was performed for each analysis. RFU; relative fluorescent units.

**Figure 4:**
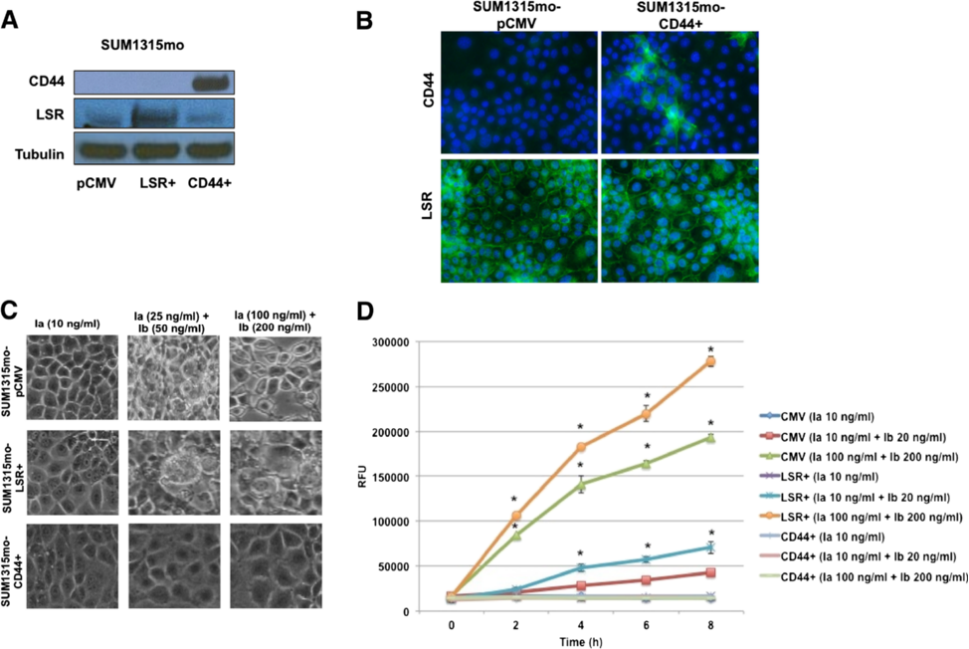
**Enhancing LSR expression increases iota cytotoxicity while reintroduction of CD44 conveys resistance in LSR**^**low**^**/CD44**^**low **^**cells.** SUM1315mo cells were stably transfected with a control plasmid (pCMV), a plasmid containing the full-length gene for LSR variant 1 (LSR+), or a plasmid containing the full-length gene for CD44 variant 1. Cells were grown to 80% confluence; lysates were isolated and analyzed **(A)** via western blot analysis using a LSR specific antibody and α-tubulin for loading control or by **(B)** immunofluorescence using CD44- and LSR-specific antibodies. DNA was stained with DAPI. **(C)** Cells were treated with Ia or Ia + Ib for 8 h in complete growth medium at 37°C. Representative images demonstrate cell rounding and detachment from tissue culture dish, indicative of cell death. **(D)** Cells death visualized in **(C)** was quantified at the indicated times via fluorescent cytotoxicity assay. *P < 0.05. A minimum of three independent experiments was performed for each analysis.

Given that introduction of LSR into LSR^low^/CD44^high^ expressing, claudin-low cell lines did not increase sensitivity to iota toxin and that CD44 can facilitate iota cytotoxicity in non-breast cancer cells [35,36], we sought to determine if iota toxin binding to CD44 was resulting in resistance to cytotoxicity. The claudin-low breast cancer cell line SUM1315mo was chosen as a model because they are LSR^low^/CD44^low^ and importantly, sensitive to iota toxin. Consistent with our prior results, stable knockdown of LSR in SUM1315mo cells (approximate 83% reduction in LSR; Figure 3A) significantly decreased toxin sensitivity in a time and dose dependent manner while overexpression significantly increased cytotoxicity compared to control cells (*P* < 0.05; Figures 3B,C and 4; cell rounding quantified in Additional file 1: Figure S1C,D). This was contradictory to our data with CD44^high^ expressing Hs578t and SUM159 cells. In the absence of CD44, overexpression of LSR in SUM1315mo cells increased sensitivity to iota toxin. This supports the hypothesis that CD44 expression in breast cancer cells may provide resistance to iota toxin. To directly test the functional role of CD44 in conveying toxin sensitivity, CD44 was overexpressed in our model (Figure 4B) and then treated with iota toxin. Reintroduction of CD44 into SUM1315mo cells indeed resulted in resistance to iota toxin (*P* < 0.05; Figure 4C, D; cell rounding quantified in Additional file 1: Figure S1E). Sensitivity of these cells was reduced to control levels even in the presence of high concentrations of iota toxin, directly demonstrating that CD44 expression conveys resistance to intoxication.

### CD44 inhibits endocytosis of iota toxin in breast cancer cells

**Figure 5:**
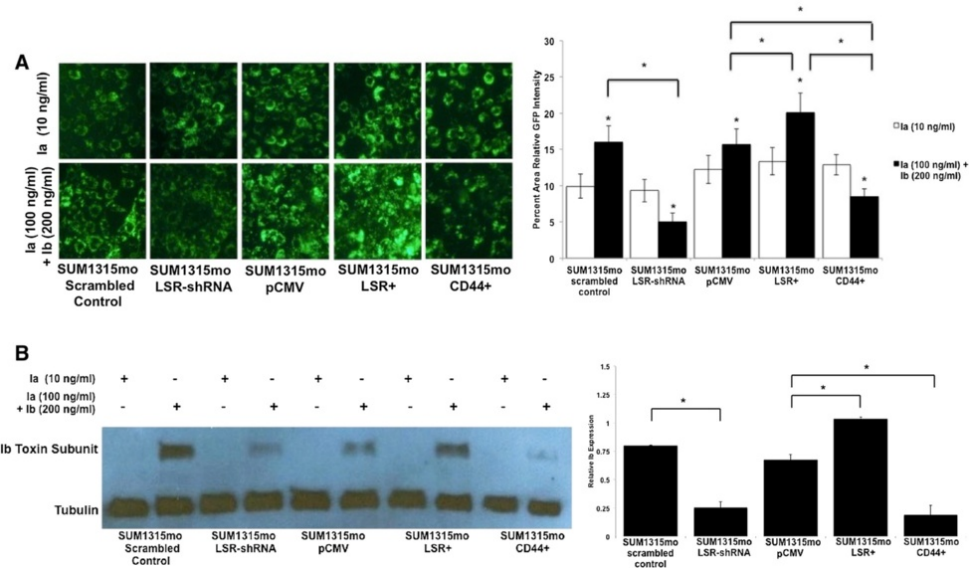
**CD44 confers resistance through inhibition of endocytosis. (A)** SUM1315mo LSR knockdown and CD44 overexpressing cells were treated with Ia only or iota toxin for 30 min, followed by lysosome imaging. A minimum of fifteen fields was viewed per treatment per experiment. **(B)** SUM1315mo LSR knockdown and overexpressing and CD44 overexpressing cells were treated with Ia or Ia + Ib for 4 h. Cells were washed, lysates were isolated and analyzed via western blot using an Ib-specific antibody; α-tubulin was used as a loading control. Representative western blot and corresponding intensity were measured via ImageJ.

While CD44 promotes iota cytotoxicity in non-breast cancer cells [35,36], our data is the first to illustrate that CD44 may actually confer resistance to iota toxin in breast cancer. To determine the mechanisms of resistance conveyed by CD44, we further investigated aspects of toxin endocytosis via lysosome formation. LSR knockout and knockin cells (SUM1315mo), as well as CD44 knockin cells were treated with Ia only (control) or a high concentration of iota toxin (Ia 100 ng/ml + Ib 200 ng/ml). When challenged with iota toxin, significantly lower levels of lysosomes were evident in LSR knockout cells compared to scramble control cells (*P* < 0.001; Figure 5A). Correspondingly, LSR-overexpression increased the number of lysosomes versus controls. Moreover, CD44-overexpression in the cells directly demonstrated a significant reduction in lysosome formation when challenged with the toxin compared to controls and LSR + cells (*P* < 0.001). These data correlate LSR expression with enhanced toxin-stimulated lysosome formation and establish a mechanism for CD44-based inhibition via lysosome formation.

To confirm our results of toxin endocytosis and incorporation into lysosomes, cells were treated with or without iota toxin, washed, and the lysates analyzed for internalized iota toxin (Ib) via western blotting. Similar to results observed with lysosome formation, knockdown of LSR significantly decreased levels of intracellular toxin compared to scrambled control, while overexpression increased toxin endocytosis (*P* < 0.05; Figure 5B). These data strongly confirm that LSR is not only a critical receptor for iota toxin cytotoxicity in breast cancer, but that it increases endocytosis of the toxin. Interestingly, reintroduction of CD44 resulted in a significant decrease in detectable Ib when compared to the control or LSR-overexpressing cells. This indicates that expression of CD44 in breast cancer cells confers iota toxin resistance by inhibiting endocytosis, a role not previously defined for CD44.

### Iota toxin has cytotoxic effects on tamoxifen-resistant breast cancer

Cellular chemotherapeutic resistance is a major factor involved in poor response and reduced survival in breast cancer patients [48]. A common and successful targeted therapy for ERα-positive breast cancers includes anti-estrogen drugs, such as tamoxifen. However, an emerging problem has been that ~33% of patients given tamoxifen therapy for five years develop recurrent tumors, and of those, 26% subsequently die [49-51]. Previous studies in our laboratory show that LSR is positively correlated with ERα expression [21]. Tamoxifen-resistant breast cancers are derived from ERα-positive tumors, and thus are likely to have high expression of LSR making them potential candidates for successful treatment with iota toxin. Ultimately, such information opens up the possibility that iota toxin represents a novel, targeted therapeutic for breast cancer.

**Figure 6:**
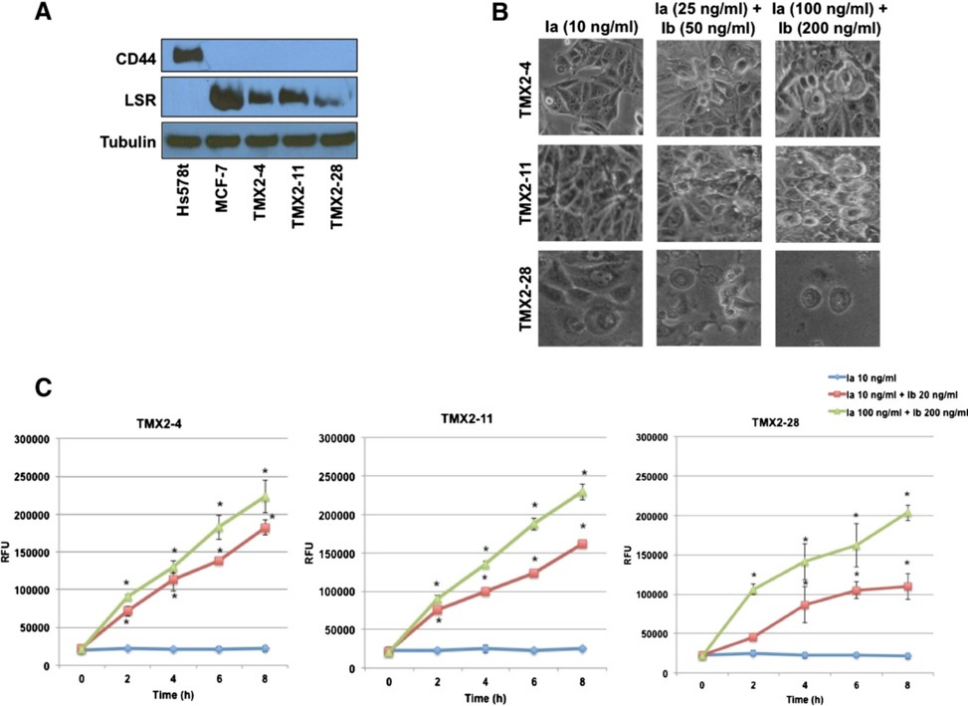
**Tamoxifen-resistant breast cancer cells expressing LSR are sensitive to iota toxin. (A)** Tamoxifen-resistant breast cancer cell lines were grown to 80% confluence, and lysates analyzed via western blot using LSR- and CD44-specific antibodies; α-tubulin was used as a loading control. **(B)** Cells were treated with Ia or Ia + Ib for 8 h and representative images demonstrate cell rounding and detachment from tissue culture dish, indicative of cell death. **(C)** Cell death was quantitated at the indicated times via fluorescent cytotoxicity assay. *P < 0.05. A minimum of three independent experiments was performed for each analysis. RFU; relative fluorescent units.

To directly evaluate iota toxin susceptibly of tamoxifen-resistant, MCF-7-derived breast cancer cell lines, ERα-positive TMX2-4 and TMX2-11, as well as ERα-negative TMX2-28, cells were assessed for LSR and CD44 expression. All three tamoxifen-resistant lines expressed LSR, but not CD44 (Figure 6A), via western blot analysis. When treated with iota toxin, all three tamoxifen-resistant lines were readily susceptible (Figure 6B,C; *P < 0.05; cell rounding quantified in Additional file 1: Figure S1F). These data suggest that iota toxin has the potential as a targeted therapy for tamoxifen-resistant breast cancers.

## Discussion

The objective of the current study was to further characterize the roles of LSR and CD44 during *C. perfringens* iota cytotoxicity on breast cancer cells. Two studies have indicated that iota toxin has the ability to bind to membrane-bound proteins, CD44 and LSR [35,36]. Complementary to the study by Papatheodorou *et al.*[35], in HAP-1 and HeLa cells, treatment of 14 breast cancer cell lines in our study show that those expressing LSR were sensitive to iota toxin. Additionally, consistent with reports in fibroblasts and hepatocytes demonstrating enhanced LSR-mediated endocytosis in the presence of oleic acid, treatment of LSR-expressing breast cancer cells with oleic acid increased sensitivity to iota toxin. However, our data presents an interesting mechanism in breast tissue that is contrary to reports in other tissues. Remarkably, breast cancer cells expressing CD44 displayed varying levels of toxin resistance. Specifically, LSR+/CD44- lines were highly sensitive, LSR+/CD44+ lines were slightly sensitive, and LSR-/CD44+ lines were resistant to the cytotoxic effects of iota toxin. Consistent with this observation, toxin sensitivity was highest in the luminal cell lines, median in basal-like, and lowest in claudin-low lines, corresponding to their reciprocal expression levels of LSR and CD44. Toxin sensitivity among the various basal-like cell lines was heterogeneous which can be attributed, in part, to the individual cell lines diverse expression of both CD44 and LSR levels. It is important to note that while our data are unlike those found in the study by Wigelsworth *et al*. [36], where they found CD44 expression promotes iota intoxication in Vero (African green monkey kidney) and human melanoma (RPM) cell cultures (*in vitro*), as well as in mice (*in vivo* lethality), the authors state that the cells they used contained LSR. They further investigated interaction between LSR and CD44 via co-precipitation experiments, showing no interaction between the proteins; however, they acknowledge that CD44 and LSR may co-facilitate entry of iota toxin into cells via an unknown mechanism. This is highlighted by the fact that the authors were unable to completely block intoxication by anti-CD44 and high amounts of toxin still cause cytotoxicity in CD44- cells [36].

Our current study suggests that the cellular response to iota toxin is tissue-specific, and for reasons not totally understood at this time. As CD44 serves many roles for a cell, and appears as many different isotypes, there clearly needs to be further study to determine more definitively the role(s) played by CD44 during iota intoxication. In fact, this current study reveals that CD44 prevents endocytosis of iota toxin and conveys cytotoxic resistance in breast cancer cells. Other studies have shown that post-translational modifications of CD44 may also affect CD44 function and endocytosis [12,40-42]. In our current study, we show that treatment with deglycosylation agents did not appreciably affect iota toxin cytotoxicity; however, other modifications unknown to us may be involved. For example, inhibition of CD44 palmitylation has no effect on CD44 binding to hyaluronan, yet there is inhibition of hyaluronan internalization [52]. Moreover, the posttranslational modifications and variants of LSR may also play a role in tissue-specific toxin endocytosis. While currently little is known about these variants and post-translational modifications of LSR, one study identifies at least one phosphorylated site (Ser^435^ within RPRARpS^435^VDAL) that affects binding of 14-3-3, a cytosolic adaptor protein involved in mediating signaling pathways by binding to phosphoserine-containing proteins [53].

Identifying the precise mechanisms of interaction between iota toxin and cell-surface proteins, as well as the subsequent downstream intracellular pathways that lead to cytotoxicity, are necessary steps in developing targeted therapies for breast cancer. Earlier studies show that in Vero cells, iota toxin enters through clathrin-independent endocytosis mechanisms, is dependent upon dynamin, and regulated by Rho-GDI [54]. Similarly, both CD44 and LSR have been proposed to be internalized via clathrin-independent mechanisms in non-breast tissues [36]. Central to our studies, breast cancer stem/tumor-initiating cells have a significantly higher rate of clathrin-independent endocytosis [55]. We have also previously shown that cells expressing high levels of LSR have enhanced cancer stem cell-like properties [21], thus collectively suggesting that iota toxin may have heightened effects upon breast cancer stem cells.

Nagahama *et al.*[56] describe the dynamics of intracellular trafficking of the Ib component of iota toxin. Through their study of MDCK cells, they found that post-internalization involves Ia escape from early endosomes into the cytosol and subsequent ADP-ribosylation of α- and β-actin. The majority of Ib goes through the endocytotic pathway into lysosomes and is degraded. A small percentage of Ib reportedly recycles back to the plasma membrane, which they suggest extends Ia entry into the target cell [56,57]. In line with this mechanism, our study revealed Ib within the lysates of LSR + cells treated with iota toxin and enhanced lysosome formation, demonstrating Ib internalization. While we did not presently analyze whether cells treated with low levels of toxin had a percentage of Ib recycled to the cell-surface, this strengthens the potential of using iota toxin as a therapeutic. Recycled Ib to the plasma membrane may further sensitize the cancer cell to a secondary toxin (Ia) treatment, thereby potentially eradicating any remaining cells.

Tamoxifen and aromatase inhibitors (AI) are commonly used to treat ERα-positive breast cancers as these therapies inhibit estrogenic signaling, ultimately leading to inhibition of cell proliferation and survival involving activated apoptotic pathways [58-61]. Tamoxifen and AI resistance are emergent clinical problems that induce phenotypic changes in tumor cells, including decreased apoptosis as well as increased proliferation and invasion [58-61]; however, the molecular mechanisms behind resistance are largely unknown. As we show in this current study, iota toxin interacts with LSR to induce cell death and LSR expression is correlated with ERα-positive breast cancers [21]. Our laboratory is currently testing the potential of iota toxin as an adjuvant therapy for women with ERα-positive, tamoxifen- and AI-resistant breast cancers. We have previously shown a multifaceted role of LSR in directing breast cancer cell behavior. For example, over-expression of LSR enhances cell proliferation and migration, as well as stimulates cancer-stem cell related properties such as survival in anchorage-independent conditions [21]. We also show that LSR expression significantly correlates with ERα expression in primary breast cancer biopsies [21]. In the current study, we show that tamoxifen-resistant breast cancer cell lines also express LSR and are sensitive to iota toxin-induced cytotoxicity. Iota toxin evidently circumvents the pro-survival mechanisms employed in anti-hormone resistant breast cancers by exploiting necrotic pathways.

Bacterial immunotoxins have been used in clinical trials to successfully treat hematological malignancies and solid tumors, as well as used as an adjuvant therapy targeting mesothelin-expressing mesothelioma, ovarian, or pancreatic cancer [62-66]. Immunotoxins derived from *Pseudomonas* exotoxin A, or plant-based ricin, subunits attached to antibody fragments have been evaluated in Phase I and II clinical trials for treating solid tumors. These trials revealed that immunotoxins could specifically target cell-surface antigens expressed at high levels in tumors. Another Phase I clinical trial, conducted by von Minckwiz and colleagues, used a single-chain immunotoxin targeting Her2 from eighteen Her2-expressing cancer patients. Intratumoral injections of immunotoxin successfully reduced tumor size [64,65]. Additionally, studies in nude mice with mesothelin-expressing tumor xenografts reveal enhanced therapeutic responses when taxon, or other cancer drugs, are administered in combination with SS1P, a high-affinity immunotoxin that targets mesothelin [66]. A Phase I trial was subsequently initiated, where mesothelin-positive and recurrent or unresectable mesothelioma, ovarian, or pancreatic cancer patients were infused with SS1P continually for ten days. The recombinant immunotoxin was well tolerated by patients and showed modest clinical benefit. Our current study revealed that iota toxin specifically targets LSR-expressing breast cancer cells and exerts a rapid cytotoxic effect. This gives iota toxin great potential to be utilized as an immunotoxin for (i) transporting foreign proteins into targeted cells, (ii) modification to increase specificity to a specific cell type, and (iii) increasing drug absorption of chemotherapeutic drugs [32,67].

Two studies with another bacterial toxin, *Clostridium perfringens* enterotoxin (CPE), reveal that CPE specifically targets claudin-overexpressing mouse NT6 fibroblasts, human colorectal adenocarcinoma (Caco-2), colon (HCT116) and mammary (MCF-7) cell lines [22,23]. The study by Walther *et al.* utilized non-viral, intratumoral *in vivo* gene transfer of CPE into mice with MCF-7 and HCT116 xenografts, resulting in reduced tumor growth compared to control groups [22]. Translational explorations of *C. perfringens* iota toxin as a chemotherapeutic are yet to be exploited to date. A study by Sakurai and Kobayashi evaluated the role of Ia and Ib subunits in guinea pigs [68]. When Ib was injected intradermally and Ia intrapertoneally, Ia was able to specifically target the Ib component in the skin resulting in localized dermonecrosis without other side effects. These studies support the feasibility of iota toxin as a specific, localized tool for drug therapy. Importantly, unlike CPE, which has the risk of eliciting its toxic effects to normal claudin expressing cells, iota toxin has had no reported effects in humans [68,69]. When combined with aforementioned results from the Sakurai and Kobayashi study, targeted therapeutics against breast cancer derived from iota toxin may provide a well-tolerated, effective alternative with lower off target effects compared to current targeted therapies.

## Conclusions

Our data presents an interesting mechanism of iota toxin cytotoxicity in breast cancer. We demonstrate that LSR is the cell-surface protein that mediates iota toxin cytotoxicity through endocytosis in breast cancer cells, and propose a novel role for CD44 as a driver of resistance towards iota toxin via inhibition of endocytotic mechanisms. Furthermore, we are the first to describe LSR expression in tamoxifen-resistant breast cancer and show the potential of iota toxin as a tool to overcome cancer-stem cell like, pro-survival mechanisms and induce necrotic cell death. Collectively, our data uniquely show that iota toxin has the potential to become an effective, targeted adjuvant therapy for breast cancer and alternative to current treatment options.

## Methods

### Cell culture

MCF-7, T47D, ZR-75-1, HCC1937, MDA-MB-468, BT-20, HCC1143, MDA-MB-231, Hs578t, BT-549, AU565, SKBR3, M99005, MCF-10AI, MCF-10AIII, and MCF-10AIV cells were obtained from American Type Culture Collection (ATCC; Manassas, VA). SUM159, SUM149, Sum190, and SUM1315mo cells were obtained from Asterand (Detroit, MI, USA). TMX2-4, TMX2-11 and TMX2-28 cells were a kind gift from Dr. Kathleen Arcaro (University of Massachusetts, Amherst). Cells were cultured according to manufacturer’s recommendations and passaged via trypsinization when approximately 80% confluent.

### Iota toxin, reagents, and toxicity testing

Iota toxin components Ia and Ib were purified as described previously [70]. For toxin sensitivity assays, cells were seeded at 1 - 3×10^4^ concentrations, enabling confluency 48 h later. Cells were then treated as either a control (10 ng Ia/ml) or with varying concentrations of iota toxin (Ia 10 ng/ml + Ib 20 ng/ml; Ia 25 ng/ml + Ib 50 ng/ml; Ia 50 ng/ml + Ib 100 ng/ml; Ia 100 ng/ml + Ib 200 ng/ml) and cultured under normal growth conditions. Observations to determine toxin sensitivity, indicated by a rounded morphology indicative of cell death, were made at 0, 1, 2, 4, 6, and 8 h post treatment. For oleic acid assays, cells were treated in the presence or absence of 0.8 mM oleic acid at 37°C, 30 min prior to added toxin. Images were obtained by Spot Advanced version 4.5 (Sterling Heights, Michigan).

### Generation of knockdown and overexpressing cell lines

RFP containing HuSH shRNA plasmids containing *Homo sapiens* LSR specific shRNA and Myc-DDK-tagged TrueORF clones of *Homo sapiens* LSR and CD44 were obtained from OriGene Technologies (cat# TF303412, RC223636, and RC221771; Rockville, MD). Cells were transfected using TurboFectin 8.0 (Thermo Scientific, Rockford, IL) according to manufacturer’s instructions. For stable transfection, cells were passaged at a 1:10 dilution into fresh growth medium containing 2.5 μg/ml Puromycin or 500–900 μg/ml of G418 (Life Technologies, Grand Island, NY). Control cells were simultaneously transfected with an empty plasmid vector and selected in antibiotic-containing medium as described above.

### Western blot analysis

Cells were lysed in RIPA Buffer (50 mM Tris Base, 150 mM NaCl, 1 mM EDTA, 1% NP40, 0.25% sodium deoxycholate) supplemented with protease and phosphatase inhibitors (Halt™ Thermo Scientific, Rockford, IL). Equal protein concentrations of total cell lysates, as determined by the Coomassie Plus Protein Assay (Thermo Scientific, Rockford, IL), were separated by SDS-PAGE. Proteins were transferred to nitrocellulose membranes (BioExpress, Kaysville, UT). Membranes were blocked in 5% non-fat milk in TBST (1.0 M Tris–HCl, 5.0 M NaCl, 0.1% Tween) for 1 h at room temperature, then incubated with primary antibody against LSR (1:750; sc-133765), HCAM (CD44; 1:500; sc-7297), E-cadherin (1:500; sc-7870; Santa Cruz Biotechnology, Santa Cruz, CA), or anti-Ib (1:1000) overnight at 4°C in TBST containing 5% BSA. Membranes were then washed and incubated with the appropriate secondary antibody conjugated to horseradish peroxidase (GE Healthcare, Piscataway, NJ) in TBST with 5% milk for 1 h at room temperature. Mouse monoclonal α-tubulin antibody was used to evaluate equal protein loading across all lanes at a 1:5000 dilution (T6199; Sigma Aldrich, St. Louis, MO). WesternBright ECL Kit (Bioexpress, Kaysville, UT) was used to detect peroxidase activity. NIH Image J64 software was used to quantify western blots.

### Immunocytofluorescence

Immunocytofluorescence was performed as previously described [71]. Briefly, cells were grown on 8-well chamber slides (Research Products International, Mt. Prospect, IL,) and fixed/permeabilized in ice-cold methanol:acetone. Following fixation, cells were blocked with 1% BSA and 5% normal horse serum in PBS, stained with the indicated primary antibody (1:100 dilution of anti-LSR, sc-133765 or anti-HCAM (CD44), sc-7297) for 1 h at 4°, washed, and then incubated for 30 min with an anti-rabbit or anti-mouse Alexa Fluor 488 secondary antibody (1:1000 dilution, Invitrogen). Coverslips were applied with ProLong® Gold Antifade Reagent and DAPI (Life Technologies). Imaging was performed on a Nikon DiaPhot microscope with digital camera and NIS-Elements 4.11.00 (Nikon Instruments Inc., Melville, NY). All cell lines and samples were obtained in compliance with the Helsinki Declaration and performed in accordance with the guidelines of the North Carolina Central University Institutional Review Board, approval 1201027.

### Glycosylation analysis

Cells were grown under normal growth conditions until approximately 70% confluent, and then serum starved overnight. Cells were subsequently treated with 25 μg/ml of either Tunicamycin (MP Biomedical LLC, Solon, OH) or Swainsonine (Calbiochem, San Diego, CA), or vehicle control. Twenty-four hours post treatment, cell lysates were collected and analyzed via western blot analysis to determine glycosylation status of LSR.

### Lysosome detection assay

Cells were grown under normal growth conditions until approximately 70% confluent. Cells were then treated as either a control (10 ng Ia/ml), or with iota toxin consisting of Ia (100 ng/ml) plus Ib (200 ng/ml), and cultured under normal growth conditions for 30 min. Following treatment, LysoTracker Green DND-26 (Cell Signaling Technology, Danvers, MA) was diluted 1:20,000 (50 nM) and added directly into growth medium, followed by imaging on a NIS-Elements 4.11.00. A minimum of three replicate wells was plated for each independent experiment, with a minimum of five fields imaged per well.

### Cell death assays

Cells were seeded at 1- 3×10^4^ concentrations, obtaining confluency 48 h later. Cells were then treated as controls (10 ng Ia/ml) or with iota toxin at low (Ia 10 ng/ml + Ib 20 ng/ml) or high (Ia 100 ng/ml + Ib 200 ng/ml) concentrations followed by culturing under normal growth conditions for 0, 2, 4, 6, and 8 h. Post treatment, cytotoxicity was determined using a CytoTox-Fluor™ Cytotoxicity Assay (Promega, Madison, WI) per manufacturer’s instructions.

### CD44 variant analysis

RT-qPCR amplification reactions were conducted in duplicate using 1X Brilliant II SYBR® Green QPCR MasterMix (Agilent Technologies, Cary, NC) in the presence of variant specific primers (800 nM each; Additional file 3: Table S2) and 40 ng of cDNA (based on total RNA) in 20 μl. A non-template reaction was used as negative control. PCR conditions consisted of denaturation at 95°C for 10 min, activation of the DNA polymerase, followed by 40 cycles of 95°C for 15 seconds and specific annealing temperatures for each splicing variant for 1 min. Melting curves were generated after amplification at 95°C for 15 seconds, 60°C for 30 seconds and 95°C for 15 seconds. All reactions were conducted in a Stratagene Mx3005P detection system (Stratagene, La Jolla, CA). Amplification efficiency of each pair of primers was calculated using standard curve dilutions and incorporated into the calculation for relative expression differences as previously described [72]. The optimal normalization factor was calculated as the geometric mean of the reference targets *B2M*, *SDHA*, *UBC* and *YWHAZ*.

### LSR variant analysis

RT-qPCR amplification reactions were conducted in duplicate using 1X Brilliant II SYBR® Green QPCR MasterMix (Agilent Technologies, Cary, NC) in the presence of optimized variant specific primers (800 nM; Supplementary Table 1) and 100 ng of cDNA (based on total RNA) in 20 μl. A non-template reaction was used as negative control. PCR conditions were the same as those used for CD44 analysis. All reactions were conducted in an Applied Biosystems’ 7500 Real Time PCR system (Grand Island, NY). Raw data were normalized to *GAPDH* and analyzed via the comparative CT (^ΔΔ^CT) method.

### Statistical analysis

To evaluate toxin sensitivity, relative percent of cell rounding was determined via visualization using light microscopy for each treatment group and statistical significances between treatment groups and across cell lines was determined by student t-test using *GraphPad Prism* version 3.02 software (GraphPad Software Inc., San Diego, CA), significance was set at P < 0.05. Differences in statistical significance between cell lines regarding CD44 and LSR expression levels (total and variants), was determined by *ANOVA* and post-hoc two-tailed comparisons. Significance was set at *P* < 0.05 and a Bonferonni correction was used to adjust the *P*-value of t-tests. Graphs were plotted in Microsoft Excel as mean ± S.D.

## Abbreviations

LSR: Lipolysis stimulated lipoprotein receptor; ERα: Estrogen receptor alpha; HER2: Human epidermal growth factor receptor 2.

## Competing interests

The authors declare that they have no competing interests.

## Authors’ contributions

JMF and BGS conceived and designed the study; KFS, DKR, MCR, and JMF performed the experiments; BGS, and JMF analyzed and interpreted data; MRP supervised the study; KFS, BGS, JMF wrote the paper. All authors read and approved the final manuscript.

## Supplementary Material

Additional file 1: Figure S1 Quantitation of iota toxin sensitivity by percent cell rounding. (A) Relative quantitation of cell rounding for Figure 1C & D, (B) Figure 2, (C) Figure 3B, (D & E) Figure 4C, (F) Figure 6B. A minimum of three independent experiments was performed for each analysis. *P < 0.05, Δ indicates *P* < 0.05 for comparison of the high toxin concentration (Ia 100 ng/ml + Ib 200 ng/ml) to the low toxin concentration (Ia 10 ng/ml + Ib 20 ng/ml). Click here for file

Additional file 2: Figure S2 Glycosylation of LSR does not play a role in toxin sensitivity. (A) MCF-7 cells were grown under normal growth conditions until approximately 70% confluence. Cells were then serum starved overnight followed by treatment with 25 μg/ml of Tunicamycin, Swainsonine, or vehicle control in normal growth medium. Twenty-four hours post treatment, cell lysates were collected and western blot analysis was performed to determine glycosylation status of LSR. (B) MCF-7 and Hs578t cells were serum starved overnight, 24 h post seeding then treated 48 h post seeding with toxin and either vehicle control or 25 μg/ml Tunicamycin. Rounding and detachment indicated cell death. Click here for file

Additional file 3: Tables S1 Iota Toxin Sensitivity of Cells Following Tunicamycin Treatment. **Table S2.** Primers used to detect splice variants. Click here for file

Additional file 4: Figure S3 CD44 variant expression does not correlate with toxin sensitivity. Relative CD44 expression was determined for seven splice variants using real time qRT-PCR. Data were normalized to the geometric mean of the reference targets *B2M*, *SDHA*, *UBC* and *YWHAZ*. A minimum of three independent experiments was performed for each analysis. Click here for file

Additional file 5: Figure S4 LSR variant expression and two LSR-related proteins, ILDR1 and ILDR2, do not correlate with toxin sensitivity. Relative LSR expression was determined for five splice variants using real time qRT-PCR as well as the two LSR-related proteins, immunoglobulin-like domain-containing receptor (ILDR) 1 and ILDR2. ILDR2 was not detected in any breast cancer samples but readily detected in monocyte cell line, THP-1 (bottom right panel; representative ethidium bromide stained DNA gel). Data were normalized to *GAPDH*. A minimum of three independent experiments was performed for each analysis. Click here for file
