# Supplementary figures and images for: Challenging the roles of CD44 and lipolysis stimulated lipoprotein receptor in conveying Clostridium perfringens iota toxin cytotoxicity in breast cancer
Source: Mol Cancer. 2014 Jul 2;13:163. doi: 10.1186/1476-4598-13-163 (PMC4086999; doi:10.1186/1476-4598-13-163)

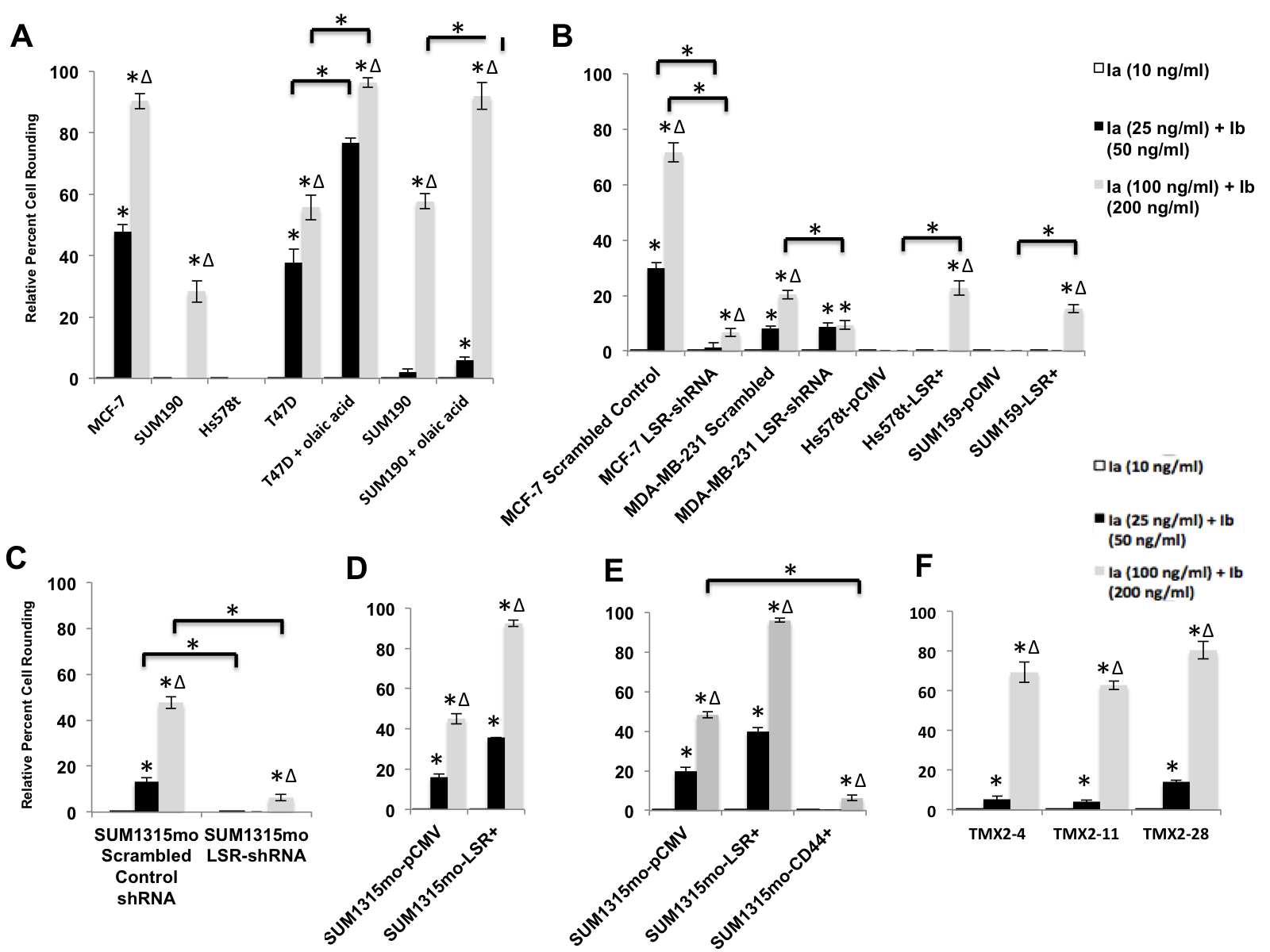

Supplement: Additional file 1: Figure S1 — Quantitation of iota toxin sensitivity by percent cell rounding. (A) Relative quantitation of cell rounding for Figure 1C & D, (B) Figure 2, (C) Figure 3B, (D & E) Figure 4C, (F) Figure 6B. A minimum of three independent experiments was performed for each analysis. *P < 0.05, Δ indicates P < 0.05 for comparison of the high toxin concentration (Ia 100 ng/ml + Ib 200 ng/ml) to the low toxin concentration (Ia 10 ng/ml + Ib 20 ng/ml). [file 1476-4598-13-163-S1.png]

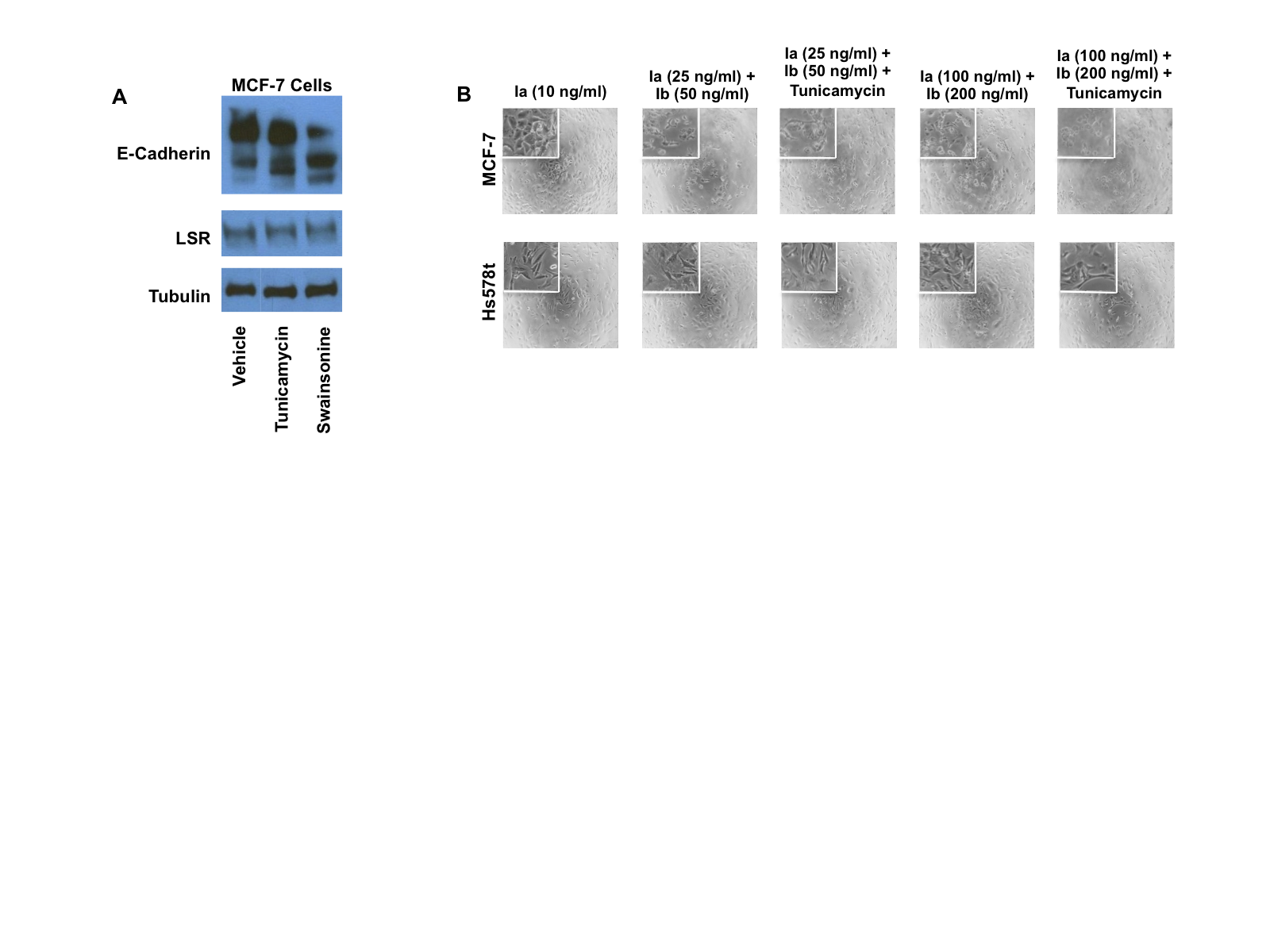

Supplement: Additional file 2: Figure S2 — Glycosylation of LSR does not play a role in toxin sensitivity. (A) MCF-7 cells were grown under normal growth conditions until approximately 70% confluence. Cells were then serum starved overnight followed by treatment with 25 μg/ml of Tunicamycin, Swainsonine, or vehicle control in normal growth medium. Twenty-four hours post treatment, cell lysates were collected and western blot analysis was performed to determine glycosylation status of LSR. (B) MCF-7 and Hs578t cells were serum starved overnight, 24 h post seeding then treated 48 h post seeding with toxin and either vehicle control or 25 μg/ml Tunicamycin. Rounding and detachment indicated cell death. [file 1476-4598-13-163-S2.png]

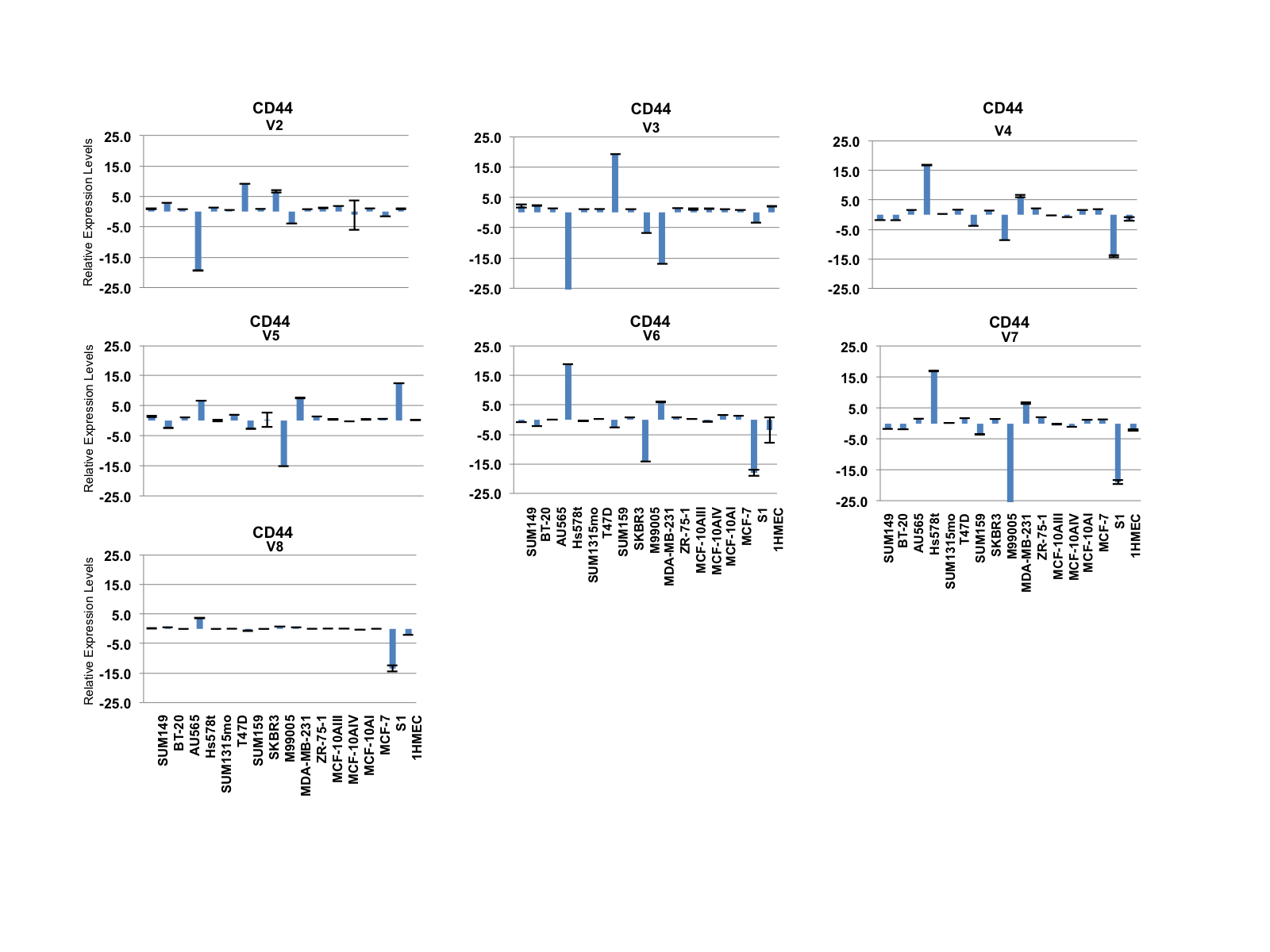

Supplement: Additional file 4: Figure S3 — CD44 variant expression does not correlate with toxin sensitivity. Relative CD44 expression was determined for seven splice variants using real time qRT-PCR. Data were normalized to the geometric mean of the reference targets B2M, SDHA, UBC and YWHAZ. A minimum of three independent experiments was performed for each analysis. [file 1476-4598-13-163-S4.png]

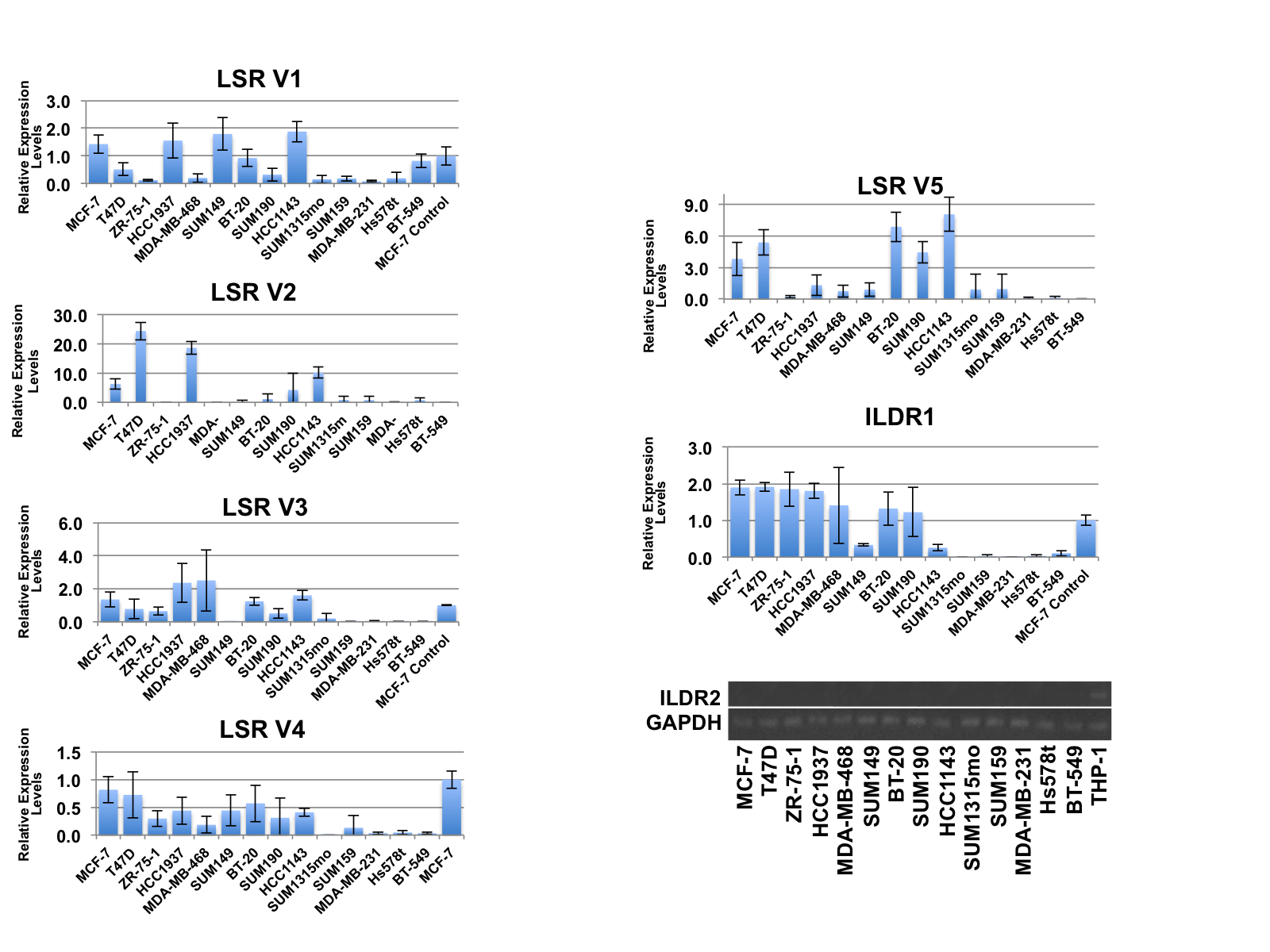

Supplement: Additional file 5: Figure S4 — LSR variant expression and two LSR-related proteins, ILDR1 and ILDR2, do not correlate with toxin sensitivity. Relative LSR expression was determined for five splice variants using real time qRT-PCR as well as the two LSR-related proteins, immunoglobulin-like domain-containing receptor (ILDR) 1 and ILDR2. ILDR2 was not detected in any breast cancer samples but readily detected in monocyte cell line, THP-1 (bottom right panel; representative ethidium bromide stained DNA gel). Data were normalized to GAPDH. A minimum of three independent experiments was performed for each analysis. [file 1476-4598-13-163-S5.png]
