# Supplementary material for: Challenging the roles of CD44 and lipolysis stimulated lipoprotein receptor in conveying Clostridium perfringens iota toxin cytotoxicity in breast cancer
Source: Mol Cancer. 2014 Jul 2;13:163. doi: 10.1186/1476-4598-13-163 (PMC4086999; doi:10.1186/1476-4598-13-163)
Supplement: Additional file 3: Tables S1 — Iota Toxin Sensitivity of Cells Following Tunicamycin Treatment. Table S2. Primers used to detect splice variants. [file 1476-4598-13-163-S3.pdf]

Table S1 Iota Toxin Sensitivity of Cells Following Tunicamycin Treatment

**Cell Line**	**Ia (10ng/ml)**	**Ia (10ng/ml) +Ib (20ng/ml)**	**Ia (10ng/ml) +Ib (20ng/ml) +Tunicamycin**	**Ia (50ng/ml) +Ib (100ng/ml)**	**Ia (100ng/ml) +Ib (200ng/ml) +Tunicamycin**
MCF-7	-	+	+	++	++
Hs578t	-	-	-	-	-
(-) Resistant, 0 to 10% cell rounding; (+) Sensitive, 11 to 50% cell rounding; (++) Highly Sensitive, >50% cell rounding. Toxin Sensitivity at 8 h post treatment					

Table S2 Primers used to detect splice variants

Gene	**Forward Sequence (5'-3')**	**Reverse Sequence (5'-3')**
**CD44 Variant 1**	GACACATATTGCTT CAATGC	CATCCCTCTTGGC CTTGGC
**CD44 Variant 2**	GAATCCCTGCTACC AGTAC	GAAAGAGACAGA CACCTCAG
**CD44 Variant 3**	GAATCCCTGCTACC AATAT	GACAGGACCTCTT TCAATG
**CD44 Variant 4**	GAATCCCTGCTACC AGAGAC	GTGGGCAGAAGAA AAAGCTAG
**CD44 Variant 5**	GATTTGAGACCTGC AGTTGC	GTGGGCAGAAGAA AAAGCTAG
**CD44 Variant 6**	GAATCCCTGCTACC AATAG	GTTATACCTCTCAT TACC
**CD44 Variant 7**	GAATCCCTGCTACC AGACA	CATCCCTCTTGGC CTTGGC
**CD44 Variant 8**	GAATCCCTGCTACC AGAGAC	CAACCTCTGGTCC TATAAG
**LSR Variant 1**	ATCGTCCTTGACTG GCTCTTC	TGTAGCCACTGCG GACTTC
**LSR Variant 2**	GTCCTTGGGAGGA CCTCAG	GAGCCAGTCTTCT ATGGGC
**LSR Variant 3**	CAGAGCTCATCGTC CTTGTGT	GGGGGCATAAATG CTGGGAA
**LSR Variant 4**	GACTGGCTCTTCGT GGTTGT	ACGTAGCAGCAGC AAGTGTG
**LSR Variant 5**	ACCATCACCGGAA TGTATGC	TCCCGAAGCAGGG GTACATA
**ILDR1**	AGCTCCCAGGTTTC ATCTTATC	GATTGGTGGTCTG GGTCATT
**ILDR2**	GATGATGTGGAGG GCAAGAA	CAAAGACCCACTC TGGCATAA
